# IoT Network for International Trade Cold Chain Logistics Tracking Based on Kalman Algorithm

**DOI:** 10.1155/2022/1608167

**Published:** 2022-09-19

**Authors:** Chao Zhang, Wei Wei

**Affiliations:** School of Management, Wuhan College, Wuhan, Hubei 430212, China

## Abstract

The application of Internet of Things technology in cold chain transportation can greatly strengthen the monitoring of all aspects of cold chain logistics, so as to promote the progress of cold chain logistics industry. In order to study this aspect, this paper chooses the key link of the Internet of Things as a breakthrough. By analyzing various aspects of the transportation process, an Internet of Things middleware framework serving cold chain transportation tracking is designed. The main products of logistics cold chain transportation are fresh agricultural products, so in order to further make the research fit the actual situation, this paper collects the logistics data and information of agricultural products through investigation and combines the core technology of agricultural products logistics with the Internet and the key technology of cold chain transportation to process the obtained information. The analytic hierarchy process and fuzzy comprehensive evaluation method are used in this process. The processing results prove the validity and rationality of the established index system. The purpose of developing the international trade management system is to enable the company to optimize its international trade management process, reduce some tedious and inconvenient manual operations, make the recording and statistics of international trade information very simple, improve work efficiency, and satisfy the information needs of various departments to enable enterprises to minimize costs, thereby enabling enterprises to obtain better economic benefits.

## 1. Introduction

The emergence of the Internet of Things platform has greatly facilitated our daily lives and has also greatly improved people's work efficiency [1]. However, it must be noted that only by complying with the rules and standards of the Internet of Things platform at this stage can the corresponding technical equipment be fully connected. Cold chain transportation is the successful application of the Internet of Things technology in the logistics platform [2]. In this link, the widespread use of sensors has greatly promoted the development of cold chain transportation. This article mainly studies how to use various types and functions. The sensors provide better service for cold chain transportation [3]. The Internet of Things middleware in the cold chain transportation link is selected as the research object. By analyzing various aspects of the transportation process, an Internet of Things middleware framework serving cold chain transportation tracking is designed [4]. In addition, in order to better deal with the communication obstacles between different sensors, and, at the same time, to filter the useless and redundant data in the sensors, we adopted D-Bus technology and designed the communication means between different sensors [5]. The focus of this article is to prove that IoT technology can optimize and enhance China's agricultural cold chain logistics. In view of this problem, the literature combined with the actual situation of the operation and development of agricultural cold chain logistics enterprises, in view of the problems existing in the enterprise process, the agricultural cold chain logistics mode starting from the enterprise was constructed [6]. The literature combines the previous research experience, uses the Internet technology, solves the practical problems in the development process of cold chain logistics, and upgrades and improves the existing logistics [7]. The international trade management system enables companies to minimize costs and better select suppliers, products, and customer information, thereby enabling companies to obtain better economic benefits [8].

## 2. Related Works

The literature has done in-depth research on cold chain logistics middleware; its purpose is to solve the problem of connection and data interaction between different sensor nodes in the cold chain tracking process in the Internet of Things environment, while ensuring the accuracy of data transmission. In the cold chain transportation link, data errors are not allowed [9]. This article adds a data processing module to the middleware of the Internet of Things, which improves the efficiency and accuracy of data collection by the middleware. The literature designed the cold chain logistics tracking middleware to solve a series of problems when collecting cold chain sensor data. The purpose of developing an international trade management system is to enable a limited company to optimize its international trade management process [10]. The international trade management system can reduce some tedious and inconvenient manual operations, make the recording and statistics of international trade information very simple, and improve work efficiency [11, 12]. The international trade management system can meet the information needs of various departments, so that companies can minimize costs and better select suppliers, products, and customer information, so that companies can obtain better economic benefits [13].

In embedded devices, embedded devices usually have many sensor nodes. It should be noted that, in these sensor nodes, the memory of each sensor node is relatively small [14]. However, according to the current state of technological development, most operating systems on the market are equipped with large-capacity storage devices, and large-capacity storage devices can promote the increase in computing speed to a certain extent. Therefore, when comparing embedded devices with other operating systems on the market, the processing speed of the embedded operating systems is much lower than other large operating systems [15, 16]. In the following description in this article, nonembedded general-purpose computer devices are collectively referred to as general-purpose devices. In daily real-life use, the general-purpose device must support the data transmission function of the near-end network, and the data transmission function enables communication and data exchange between the sensor node and the general-purpose device [17]. D-Bus technology is an end-to-end communication technology, which is used for each sensor and general device in the near-end network to ensure that the communication and data transmission between the sensor node and the general device can be carried out normally [18].

## 3. Construction of IoT Sensor Network for Cold Chain Logistics Tracking

### 3.1. Overview of Related Technologies of IoT Middleware

REST refers to the state transfer of the presentation layer, which means that the relevant data of the resource network management center is transferred in a certain way or in a certain state. The main manifestations of the resource representative data are JSON, XML, etc., and the state change is through HTTP verb implementation.

In a period when Internet technology and science and technology were not developed, there was a problem of low penetration rate of mobile device terminals. Therefore, in order to save resources and reduce expenses, the client and server are usually combined together. However, in recent years, science and technology have continued to develop, and the penetration rate of the Internet and the Internet of Things has been rising, and various types of Client service plug-ins have appeared. Therefore, REST can provide services for various types of platforms through related service plug-ins.

In the process of REST providing services for various platforms, REST is always in the stateless constraint stage. Therefore, when the server and the client use the REST framework for interactive communication, the server does not need to know the usage status of the client. Moreover, in the process of interaction, there will be no information loss, and the server and the client can clearly identify and judge the received information.

REST is in the stateless constrained stage. It means that when REST provides services, each web service in REST is in an independent state, and the server does not save the relevant state information of the client.

The REST system conducts standard state interaction through its own resources. Therefore, when REST provides services, it does not require a specific interface as an intermediary.

Four basic HTTP verbs are used in the REST system to interact with resources, as shown in Table 1.

Using the REST architecture can make full use of the various functions brought by the HTTP protocol and make the software architecture design clearer and maintainable.

Hidden Markov model is a kind of probability model, which expresses the sequentiality of a set of random processes through probability description. In the hidden Markov model, the most important component is the Markov chain. In the actual application process of the hidden Markov model, the real state of the location is represented by the hidden Markov chain.

(1) $$\begin{array}{c} P\left(X_{n}=x\left|X_{0},X_{1},X_{2},\ldots X_{n-1}\right.\right)=P\left(X_{n}\left|X_{n-1}\right.\right)\text{.} \end{array}$$

Assume that *Q* is the set of all states, and *V* is the set of all observed states.

(2) $$\begin{array}{c} Q=\left\{q_{1},q_{2},\ldots ,q_{N}\right\},V=\left\{v_{1},v_{2},\ldots ,v_{M}\right\}\text{.} \end{array}$$

*I* is the state sequence of length *T*, and *O* is the corresponding observation sequence.

(3) $$\begin{array}{c} I=\left\{i_{1},i_{2},\ldots ,i_{T}\right\},V=\left\{o_{1},o_{2},\ldots ,o_{T}\right\}\text{.} \end{array}$$

The state transition probability matrix is as follows:

(4) $$\begin{array}{c} A={\left[a_{ij}\right]}_{N*N}\text{.} \end{array}$$

Among them,

(5) $$\begin{array}{c} a_{ij}=P\left(i_{t+1}=q_{j}\left|i_{t}=q_{i}\right.\right),1\leq i,j\leq N\text{.} \end{array}$$

The observation probability matrix is

(6) $$\begin{array}{c} B={\left[b_{j}\left(k\right)\right]}_{N*M}\text{.} \end{array}$$

Among them,

(7) $$\begin{array}{c} \pi_{i}=P\left(i_{1}=q_{i}\right),1\leq i\leq N\text{.} \end{array}$$

The hidden Markov model is represented by a ternary symbol:

(8) $$\begin{array}{c} \lambda =\left(A,B,\pi \right)\text{.} \end{array}$$

The Kalman algorithm is the most commonly used estimation algorithm. The Kalman algorithm has the characteristics of small calculation requirements and good recursion in the actual application process. Therefore, since the Kalman algorithm was proposed, it has been widely used, and the Kalman algorithm has gradually developed into an optimal estimation algorithm. The typical use of Kalman algorithm is to smooth noisy data. Kalmar filter is an accurate reasoning algorithm, which is similar to Bayesian model, but there are certain differences between the two. The latent variable of Kalman filter is always in a continuous state in the state space.

Kalman filter is mainly used in solving random linear discrete systems and parameter estimation. In the actual application process, the steps of using the Kalman filter are divided into estimation and correction. Estimation refers to constructing a priori estimation of the next state based on the current prior state value and then after a reasonable calculation. Correction refers to the use of parameter update equations to check the previously constructed prior estimates. The correction process is mainly a feedback process.

The time update equation and state update equation of the discrete Kalman filter are as follows:

(9) $$\begin{array}{c} \begin{array}{c} {\hat{X}}_{k}^{-}=A{\hat{X}}_{k-1}+B{\hat{U}}_{k-1}\\ {\hat{P}}_{k}^{-}=A{\hat{P}}_{k-1}A^{T}+Q\\ K_{k}=P_{k}^{-}H^{T}{\left(HP_{k}^{-}H^{T}+R\right)}^{-2}\\ {\hat{X}}_{k}={\hat{X}}_{k}^{-}+K_{k}\left(Z_{k}-H{\hat{X}}_{k}^{-}\right)\\ P_{k}=\left(I-K_{k}H\right)P_{k}^{-}\text{.} \end{array} \end{array}$$

### 3.2. IoT Middleware Architecture for Cold Chain Logistics Tracking

**Figure 1:**
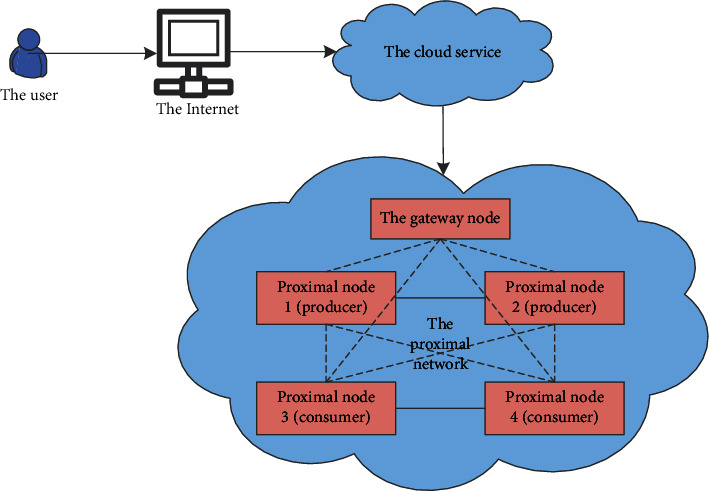
Middleware remote access network architecture.

This article assumes that each node with middleware installed is peer-to-peer; that is, each node can be either a consumer or a producer. Consumers remotely call the near-end network to find the required nodes and obtain services, and the provider nodes have unique IDs to facilitate the calls of consumers.  Figure 1 describes how external devices access specific services required in a near-end network.

**Figure 2:**
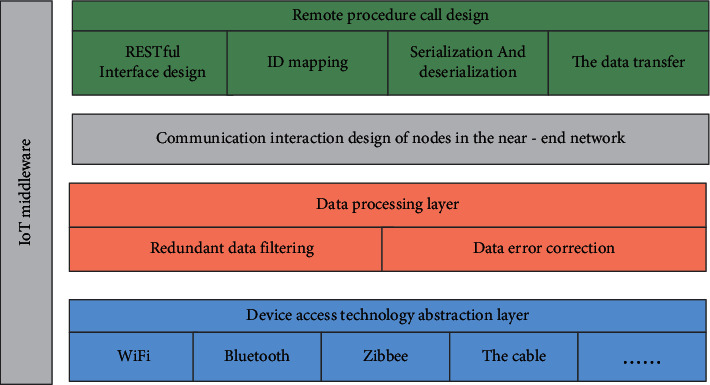
Middleware architecture block diagram.

Based on the design idea of this article, in the cold chain traceability environment, the IoT middleware needs to trace all the data in its environment and also provide services for each node of the sensor. The IoT middleware can schedule the location and location of sensor nodes in real time. The current data transmission method uses the above functions to complete data collection and data error correction. In summary, the detailed system architecture diagram of the IoT middleware is shown in Figure 2.

According to Figure 2, the IoT middleware proposed in this article mainly does the following work:

(1) Access technology abstraction layer: when sensor nodes choose different transmission methods, there is no need to write communication code.
(2) Data processing: including filtering of redundant data and error correction of data with strict requirements.
(3) Remote call node service: the method executed by remote call runs in the remote node. Unlike local call, the remote node service runs in a different process. To communicate with it, the following problems need to be solved:
  - ID mapping: because the function pointer cannot be used like a local call, the corresponding method in each node must have a corresponding ID to implement the corresponding method.
  - Serialization and deserialization.
  - Data transmission.
(4) Different node communication design: introduce D-Bus technology, connect each sensor node to the session bus, connect general equipment to the session bus and system bus, and then use the system bus daemon to communicate with the sensor nodes.

In the process of cold chain traceability, a large number of sensor nodes are used, and these sensors will generate a large amount of real-time data. However, in actual use, due to various problems such as poor network quality, some error data will be generated, resulting in redundancy of data information; due to the existence of error data, the processing efficiency of the system is greatly reduced. Therefore, how to deal with these wrong data has become the focus of the next research. Because the cold chain link requires data to have high accuracy, data error correction is required, and unnecessary network expenses can be reduced through data error correction.

#### 3.2.1. Redundant Data Filtering

In the Bloom Filter-based filtering algorithm, the system adds a timestamp attribute to each data that reaches the data processing layer and sets the time threshold between two data receptions. When the time difference between two adjacent data received exceeds the threshold, or the time difference is less than the threshold, but the data is the same, it is determined as redundant data; otherwise, it is determined that the remaining data is nonredundant data.

**Figure 3:**
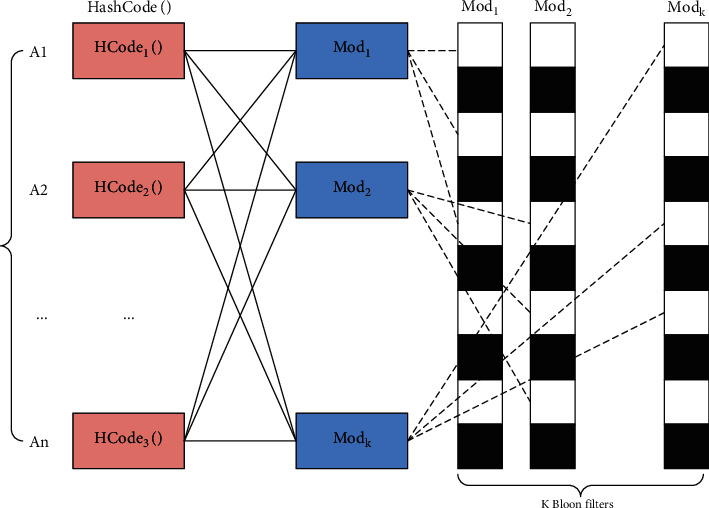
Bloom filter filtration diagram.

Set Terminal ID to represent the ID of the sensing device, which has unique properties, and Send Time represents the timestamp when data is sent to the data processing layer, and the set time interval between adjacent data is the Time Threshold. The specific schematic diagram is shown in Figure 3.

Then, establish the following rules:

- Check the parity bit of the data to determine whether the data is distorted.
- After receiving the data, the data processing layer determines whether there is a terminal identification code in the sensor device information corresponding to the receiving time point.
- Judging whether the new data value of the same terminal identification code in rule (b) is the same: if they are the same, it is judged as duplicate data; if they are different, the original data is updated.
- The old data and the same data are judged as redundant data, and the redundant data is directly discarded.

Therefore, when the data processing layer receives data, the system processing steps are as follows:

  (a1) Determine whether the data is distorted by the check bit, and discard it if distorted.
  (b1) If the received data is not distorted, judge whether there is a device ID in the sensor device information corresponding to the receiving time point. If not, directly add the data to the data structure; if there is, determine whether it is old data. If it is old data, discard it; if it is new data, go to step (c).
  (c1) Determine whether the added data is duplicate data; if it is duplicate data, discard it.

Sensor data error correction: after the transmission data is obtained, due to the influence of factors such as environment and equipment, in the process of data display and processing using the obtained data, error data that obviously does not conform to the logic and the scene will be found. Therefore, a corresponding data error correction module needs to be added to the middleware of the Internet of Things. The data error correction is performed through the data error correction module, and the error data is eliminated, thereby improving the accuracy of the collected data.

#### 3.2.2. Design and Implementation of Middleware Remote Procedure Call

In the cold chain traceability link, users of sensor nodes and middleware can call the internal data of the system at any time and can provide users with corresponding service requirements by calling the data.

*(1) REST Interface Design*. It is extremely important to design a unified style interface, which is also clear for later maintenance. The corresponding verbs and resource operations are shown in Table 2.

According to the above principles, the design of the cold chain link interface is as follows:

1. GET: get the temperature of a specific node
2. POST: update the temperature of the node (error correction operation, etc.)

**Figure 4:**
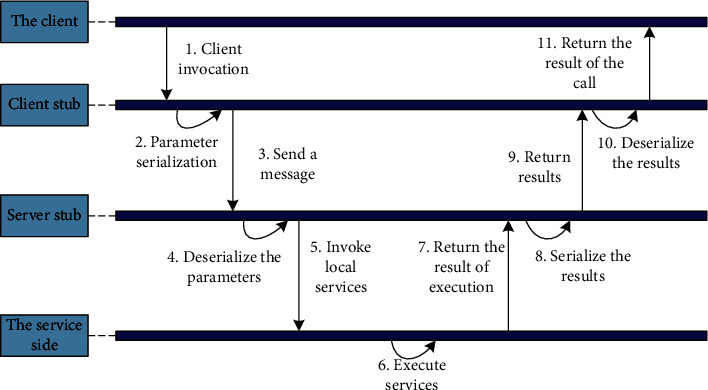
Serialization and deserialization diagram.

When using the local method for data processing, you only need to add the data to be processed to the relevant stack, and the local method can automatically call the relevant parameters to process the relevant data. In the method of using remote calls, the programming language used by the client and the server may be different, and the processing process may not be at the same stage. Therefore, the memory transfer method cannot be used for data processing. Therefore, the client needs to convert the parameters and data it wants to transmit into a binary byte stream and then send it to the server. Finally, the server converts the received binary byte stream into a coded form that it can read. In the above operations, the process of converting parameters and data into a binary byte stream is called serialization, and the process of converting a binary byte stream into a readable encoding form is called deserialization. In the actual application process, whether the client transmits parameters and data to the server, or the server transmits parameters and data to the client, both of them need to undergo the serialization and deserialization processes. The related schematic diagram is shown in Figure 4.

In the actual application process, the programming languages used by different servers and different clients are not the same, and different programming languages will cause differences in data structures, and differences in data structures will lead to differences in the representation of binary structures. If different, the client and server need to specify some transmission protocols when performing serialization and deserialization operations. Currently, common transmission protocols include XML, JSON, and Thrift. However, the XML transfer protocol has certain disadvantages. Since the amount of information transmitted between the sensor node and the client is not large, the resulting byte stream will be lengthy and complicated when the XML protocol is used for serialization. In contrast, the JSON protocol is better in the process of use. Compared with the XML protocol, the JSON protocol uses the data processed by the JSON protocol to have a simpler data format, faster parsing density, and better readability. The most important point is that, after serialization using the JSON protocol, the resulting file size is smaller. Therefore, in actual applications, in order to ensure the conciseness of the obtained data, we often use the JSON protocol as an intermediate protocol when performing serialization and deserialization operations.

In the actual use, you will find that the computing power of embedded devices will be affected by the memory of the sensor node. If the memory of a sensor node is larger, its computing power will be stronger. Similarly, if the memory of a sensor node is smaller, the computing power of the embedded device is smaller. Therefore, when the sensor node is in use, it is only introduced into the session bus to reduce expenses through such measures. General-purpose devices are different from sensor nodes because general-purpose devices need to serve the entire system at all times. Therefore, in the process of use, general-purpose devices are introduced into the system bus and the session bus. The system bus will automatically start when the general-purpose device is running and shut down when the general-purpose device is closed. In the system bus, the bus daemon can communicate with other processes in the system during the running process, and the sensor node is running. In the process, different sensors can use different session buses to access the system bus. Therefore, sensor nodes can send signals by using the session bus and then connect with each process. The specific steps are divided into the following: (1) the node first sends the target address to the general-purpose device to request access to the system bus. (2) The system bus processes the request information relayed by the general-purpose equipment and forwards the request information to the logo node. (3) The slogan node responds to the received information, and then the daemon sends the response information back to the node.

**Figure 5:**
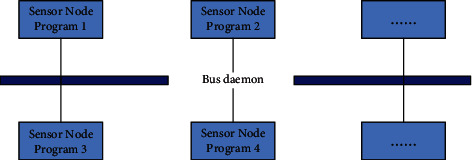
Schematic diagram of communication between near-end nodes.

Through the specific communication steps, it can be seen that the communication between the node and the system needs to pass through the daemon of the system bus, which means that when the sensor node communicates with the system, the daemon of the system can play an intermediary role, and at the same time, it can ensure the security of messages and the schematic diagram of communication between nodes, which are shown in Figure 5.

In summary, sensor nodes can communicate with general-purpose devices. In the process of connection and communication, the daemon acts as an intermediary to ensure the security of information in this way.

### 3.3. Research on Data Error Correction Technology of Cold Chain Logistics Middleware

The Kalman filter is suitable for the estimation of dynamic data. The optimal state is given by estimating the dynamic data. Because there is noise in the dynamic data, the data measured by the sensor is not accurate, so the Kalman filter is used to filter out the noise and estimate the true state value of the data.

During data calculation, vector data such as temperature and humidity can be converted into scalar data.

The predicted deviation of temperature type is

(10) $$\begin{array}{c} T_{pe}=\sqrt{T_{oe}^{2}+T_{ue}^{2}}\text{.} \end{array}$$

In a cold chain vehicle, first assume that the temperature at time *K* and at time *K* − 1 are the same. At this time, calculate the Kalman gain:

(11) $$\begin{array}{c} Kg=\frac{T_{pe}^{2}}{T_{pe}^{2}+T_{se}^{2}}\text{.} \end{array}$$

And estimate the temperature at time *K*:

(12) $$\begin{array}{c} T_{k}=T_{k-1}+Kg*\left(T_{s}-T_{k-1}\right)\text{.} \end{array}$$

Solve the optimal deviation at time *T*_*k*_:

(13) $$\begin{array}{c} T_{oe}=\sqrt{\left(1-Kg\right)*T_{pe}^{2}}\text{.} \end{array}$$

Iteratively process the calculated temperature at *T*_*k*+1_ and the measured temperature to obtain more realistic temperature data.

It should be noted that if the monitored data is very far away from the corresponding road section, it can be determined with a high probability that the detected location point is not on the road section. However, if the GPS signal has a large error, the above situation will also occur. Therefore, under this background, this article gives a related optimization plan, through which this kind of situation can be avoided, thereby improving the accuracy of trajectory correction. The relevant calculation formula is as follows:

(14) $$\begin{array}{c} p\left(y_{x}\left|p_{x}=p_{i}\right.\right)\text{.} \end{array}$$

In the calculation process, taking into account the GPS noise during the transportation process, the value of the element in the observation probability matrix is set as

(15) $$\begin{array}{c} bi=p\left(y_{x}\left|p_{x}=p_{i}\right.\right)=\frac{1}{\sqrt{2\pi \sigma }}e^{{\left(\frac{\left\|y_{x}-p_{i}\right\|gc}{\sqrt{2}\sigma }\right)}^{2}}\text{.} \end{array}$$

Among them, *σ* is the standard deviation of GPS measurements. When solving the state transition probability function, there is no need to consider the positioning information relationship between the devices; only the information between the real positions is considered. If the distance between the two candidate real positions is shorter, the probability that the two candidates actually undergo a state transition is also greater. Based on the above situation, it can be seen that the state transition probability is proportional to the distance between candidate points.

The relevant calculation formula is as follows:

(16) $$\begin{array}{c} aij=p\left(p_{x+1}=p_{j}\left|p_{x}=p_{i}\right.\right)\propto e^{-\beta d_{ij}}\text{.} \end{array}$$

The initial state probability of the vehicle trajectory model can be expressed as

(17) $$\begin{array}{c} p\left(y_{1}\left|p_{1}=p_{i}\right.\right)\text{.} \end{array}$$

The path solved by the Vibit algorithm will correspond to a hidden state sequence. Its iterative principle formula is as follows:

(18) $$\begin{array}{c} \delta_{t+1}\left(i\right)=\max_{i_{1},i_{2},\ldots ,i_{t-1}}P\left(i_{t+1}=i,i_{t},\ldots ,i_{1},o_{t+1},\ldots ,o_{1}\left|\lambda \right.\right)\max_{1\leq j\leq N}\left[\delta_{t}\left(j\right)a_{ji}\right]b_{i}\left(o_{t+1}\right),i=1,2,\ldots N;t=1,2,\ldots ,T-1\text{.} \end{array}$$

Then, in time *t*, the maximum probability node of a single path is

(19) $$\begin{array}{c} \Psi_{t}\left(i\right)=\underset{1\leq j\leq N}{argmax}\left[\delta_{t-1}\left(j\right)a_{ji}\right]b_{i}\left(o_{t+1}\right),i=1,2,\ldots N\text{.} \end{array}$$

In the actual survey process, the original data was obtained through questionnaire surveys, and the corresponding weight judgment matrix was determined by comparing the importance of indicators. First-level index weight judgment matrix is shown in Table 3. IOT organizational structure U1 weight judgment matrix is shown in Table 4. Secondary index score is shown in Table 5.

1. Data that requires stable temperature and humidity:
2. In the process of data processing, the temperature of the cold chain vehicle is set to *T* = 5°C, and then the values of Tpe, Kg, Tk, and Toe are calculated according to the smooth data error correction algorithm of Kalman filtering.
3. Latitude and longitude:

Choose about 500 anchor points, and the time interval between each anchor point is *s* to obtain ten trajectory data sets. The time is stored in a unix timestamp format for convenience. Some data are shown in Table 6.

## 4. Demand Analysis and System Realization of International Trade Management

### 4.1. International Trade Management Demand Analysis

Through the LAMP trade management system, users can conveniently and quickly query relevant data and information, while also reducing management costs, making enterprise management systematized and accurate. The system management module is composed of many management subsystems, and the administrator manages the whole by manipulating the subsystems. The management system mainly provides managers with the following two aspects of help: first, manage employee information. Second, the system administrator can back up, restore, and manage the information in the database. At the same time, the system also restricts the administrator's authority and functions to a certain extent to ensure the safety of the entire system, thereby providing convenience for international trade management activities.

The purchasing management module of the system is usually controlled by purchasing personnel. The specific business process is as follows: first, the purchasing personnel determine the purchase list according to the actual situation, and the management and modification of the list are controlled by the purchasing personnel. After receiving the purchase list, the purchasing department confirms the content of the purchase order. The system combines the data and information of each supplier in the database to make a comprehensive judgment and gives the most cost-effective supplier information. The purchasing department conducts actual negotiation with the supplier based on the information provided by the system and then confirms the purchase.

The inventory management module provides support for warehouse administrators in the following activities: product storage, product delivery, supplier information management, and product viewing. The data analysis module is solely responsible for the corporate accounting. The accountant can perform the following activities in the system data analysis module: manage loss information, manage financial information, and query out-of-stock information.

### 4.2. Implementation of an International Trade Management System

System management mainly includes four aspects: data security, administrator, user management, data backup and recovery, and authority management. The system has a strong ability to protect information. When the computer is running the system, if the user leaves in the middle, the system will automatically lock the computer to protect the important information from being read by others. At the same time, the system gives the administrator an important authority, which is to modify the login restrictions of ordinary users.

The procurement management module is the most critical and core part of the entire system. This module is responsible for purchasing planning, expediting, checking, and receiving warehouse, settlement and payment, etc. At the same time, the procurement management module realizes a global material supply cycle. The main process of procurement management is as follows: the buyer determines the purchase list in the purchase interface according to the actual situation, the system sends the purchase information to the supplier, and then the supplier is responsible for transporting the purchased materials. After the materials arrive, the inspection department will proceed according to the purchase list acceptance. Therefore, the inventory management module must have three functions: inbound acceptance, outbound acceptance, and raw material inventory inquiry.

Sales are inseparable from any commodity, so the sales management module is also an extremely important part of the system. The sales management module provides sales forecasts, sales plans, and sales contracts for corporate sales personnel, as well as a series of information about product sales to improve the core competitiveness of the company in the commercial field. The data analysis module uses a series of high-tech methods such as big data, the Internet, and intelligent algorithms to analyze the current operating conditions and competitive environment, while giving correct judgments. The data analysis module can truly understand the market and can respond to market changes in a timely and effective manner.

## 5. Conclusion

The rapid development of the Internet of Things technology makes the Internet of Things platform based on the Internet of Things technology spring up like mushrooms. The emergence of the Internet of Things platform has greatly facilitated our daily lives and has also greatly improved people's work efficiency. However, at present, there are multiple communication standards and mechanisms in the Internet of Things platform. How to quickly and conveniently exchange information between different devices is a key issue that we must consider in the research process of Internet of Things middleware. The article selects the Internet of Things middleware in the cold chain transportation link as the research object and designs an Internet of Things middleware framework for cold chain transportation tracking by analyzing various aspects of the transportation process. In addition, in order to better deal with the communication obstacles between different sensors and filter the redundant data in the sensors, we adopted D-Bus technology and designed the communication means between different sensors. The development and utilization of the international trade management system can reduce some tedious and inconvenient manual operations, make the recording and statistics of international trade information very simple, improve work efficiency, and, at the same time, meet the information needs of various departments, so that enterprises can minimize cost and better select suppliers, products, and customer information, so that enterprises can obtain better economic benefits.

## Figures and Tables

**Table 1:** HTTP verbs in REST.

Verb	Operating
GET	Retrieve a specific resource or collection of resources
POST	Create a new resource
PUT	Update a specific resource (by id)
DELETE	Delete specific resources by id

**Table 2:** Verb resource operation table.

Verb	Operating
GET	Get resources (by id)
POST	New resource (by id)
PUT	Update resource (by id)
DELETE	Delete resource (by id)

**Table 3:** First-level index weight judgment matrix.

First level indicator	U1	U2	U3	U4
Organizational structure of the internet of things U1	1	1/5	1/3	3
IoT benefits u2	5	1	3	7
RFID technology U3	3	1/3	1	9
GPS/GIS technology U4	1/3	1/7	1/9	1

**Table 4:** IOT organizational structure U1 weight judgment matrix.

Organizational structure of the internet of things U1	U11	U12	U13	U14
Internet of things U11	1	1/6	1/5	1/3
Internet of things technology U12	6	1	3	2
Talent U13	5	1/3	1	3
System flexibility U14	3	1/2	1/3	1

**Table 5:** Secondary index score.

Impact level\secondary index standard	Very satisfied	Quite satisfied	General	Not so satisfied	Very dissatisfied
IoT devices	0.2	0.3	0.3	0.1	0.1
Internet of Things technology	0.3	0.4	0.2	0.1	0.0
Talent allocation	0.1	0.2	0.4	0.2	0.1
System flexibility	0.2	0.4	0.2	0.1	0.1
Business operations	0.1	0.4	0.2	0.1	0.2
Total operating cost	0.1	0.5	0.2	0.1	0.1
Service quality	0.2	0.1	0.1	0.3	0.3
Information processing level	0.4	0.3	0.1	0.1	0.1
Information collection	0.2	0.5	0.2	0.1	0.1
Agricultural product traceability	0.3	0.4	0.1	0.1	0.1
Temperature and humidity control	0.4	0.3	0.2	0.1	0.0
Information security	0.2	0.3	0.2	0.2	0.1
Distribution vehicle skeleton	0.4	0.3	0.2	0.1	0.0
Delivery vehicle positioning	0.2	0.5	0.1	0.1	0.1
Warehouse location setting	0.3	0.3	0.2	0.2	0.0
Comprehensive map query	0.0	0.2	0.4	0.2	0.2

**Table 6:** Partial data of trajectory data set.

Coordinate system	Longitude	Latitude	Positioning time (unix timestamp)	Speed (km/h)
BD09	118.9430657846885	32.117O51885385809	1544569487	32.09
BD09	118.94337778538228	32.1I619665702898	1544369492	32.22
BD09	118.94353585 19896	32.115779705285238	1544569497	32.84
BD09	118.94582966804825	32.115190979212808	1544569502	36.26
BD09	118.9441687O733784	32.11463515998318	1544569507	38.61
BD09	118.94441540247716	32.11411700783336	1544369512	39.21
BD09	118.94471602414238	32.11361997022351	1544569517	40.09
BD09	118.945O6685865O49	32.113045092045648	1544569522	42.07
BD09	118.94533914710071	32.11259178169525	1544369527	41.83

## Data Availability

The data used to support the ﬁndings of this study are available from the corresponding author upon request.
